# Effect of 5/6 Nephrectomized Rat Serum on Epithelial-to-Mesenchymal Transition In Vitro

**DOI:** 10.3109/0886022X.2011.585416

**Published:** 2011-06-10

**Authors:** Zhaoyu Lu, Yuansheng Xie, Xusheng Liu, Shaoyuan Cui, Yuchi Wu, Cun Cai, Lei Zhang, Xiangmei Chen

**Affiliations:** 1 Department of Nephrology, Chinese PLA General Hospital, State Key Laboratory of Kidney Disease, Beijing, PR China; 2 Department of Nephrology, 2nd Affiliated Hospital of Guangzhou University of Chinese Medicine, Guangzhou, PR China

**Keywords:** 5/6 nephrectomized rat serum, epithelial-to-mesenchymal transition, E-cadherin, ZEB1, β-catenin

## Abstract

*Objective:* To investigate whether the 5/6 nephrectomized (5/6Nx) rats’ 12-week serum could lead to tubular epithelial-to-mesenchymal transition (EMT) and its molecular mechanism, so as to probe the potential stimulation from circulation in chronic progressive kidney disease. *Methods:* A total of 24 Sprague Dawley (SD) rats were randomly divided into two groups: sham operation group (sham group) and 5/6Nx group. Rats were killed 12 weeks after surgery to obtain 5/6Nx rats’ 12-week serum. Then we detected the expression of E-cadherin in renal tubular epithelial cells of the remaining kidney and we investigated whether the 12th week serum of 5/6Nx rats could cause HK-2 (human kidney proximal tubular cell line) cells to transdifferentiate into fibroblasts. *Results:* Our data confirmed that E-cadherin expression decreased significantly in the remaining kidney at 12 weeks, and the 5/6Nx rats’ 12-week serum could suppress E-cadherin protein and mRNA expression (*p* < 0.05). We also found that the 5/6Nx rats’ 12-week serum could upreg-ulate ZEB1, β-catenin, and wnt3 protein expression (*p* < 0.05). *Conclusions:* Our results demonstrated that the 5/6Nx rats’ 12-week serum could suppress the expression of E-cadherin in HK-2 cells. It was partially through modulating the increase of ZEB1. The loss of E-cadherin could lead β-catenin to localize to the cytoplasm and nucleus, and feed into the Wnt signaling pathway. It means that the pathogenic serum in chronic kidney disease (CKD) plays an important role in the loss of renal function and turns to be a new avenue of research with potential clinical implications.

## INTRODUCTION

Renal fibrosis is the principal process underlying the progression of chronic kidney disease (CKD) to end-stage renal disease (ESRD).^1^ It involves glomeru-losclerosis and tubulointerstitial fibrosis. However, tubulointerstitial fibrosis has evolved as the most consistent predictor of an irreversible loss of renal function and progression to ESRD. Recent studies have demonstrated that a critical step in the pathogenesis of tubulointerstitial fibrosis is epithelial-to-mesenchymal transition (EMT),^2^,^3^ whereby renal tubular epithelial cells change phenotypically and functionally into myofi-broblasts. Activation of tubulointerstitial myofibroblast leads to the production of excessive extracellular matrix with a predominance of interstitial collagens and plays a critical role in the progression of CKDs.^4^

A number of factors may initiate EMT in kidney disease. Much is known about the molecular background of fibrosis that the growth factors and cytokines, including transforming growth factor-β (TGF-β), fibroblast growth factor (FGF), interleukin-1 (IL-1), epidermal growth factor (EGF), and angiotensin II,^5^–^9^ prompt fibroblasts or transdifferentiate kidney cells to release collagen and matrix components.^10^ However, the growth factors and cytokines mainly take effect through local autocrine and paracrine.

In CKD patients, with the decrease in glomerular filtration rate, the excretion of metabolites fails. The body also undergoes oxidative stress, carboxyl shock, micro-inflammation, and other pathological and physiological changes,^11^,^12^ where some metabolic toxins are accumulated in the body. The endocrine system in CKD patients is also abnormal, such as abnormal circulating renin-angiotensin-aldosterone system (RAS) together with a variety of vasoactive substances mediating renal injury.^13^ Therefore, it is of great importance to probe the stimulation from circulation in chronic progressive kidney disease.

The 5/6 nephrectomized (5/6Nx) rat model is the most typical model for CKD study. After 5/6 nephrectomy, the remnant kidney initiates inflammation to repair the injured kidney. Then, the remnant kidney unit has a disturbance in hemodynamics, with high perfusion, high filtration, and high pressure. This disturbance results in scar repair of the injured nephrons and compensatory hypertrophy, hyperplasia, and sclerosis of the remnant nephrons. Ultimately the remnant kidney manifests as progressive renal dysfunction and accumulates metabolic toxins.^14^

Prior to our experiment, we took an overall review of the dynamic changes of renal pathological morphology in the 5/6Nx rat model. It was shown that glomerular sclerosis and tubulointerstitial fibrosis, which were similar to the pathologic situation of the majority of CKD patients, were hardly identified at the 8th week after nephrectomy, but were apparent at the 12th week.^15^ We can assume that the experimental kidney compensated for 8 weeks, but never for 12 weeks. It should be worse at the 16th week. Our research aimed to explore any potential pathologic stimulation from circulation system at an early stage, so we employed the serum at the 12th week as the pathological stimulation. EMT is believed to be a promising mechanism, so we chose it as the entry point of research, by exerting the 5/6Nx rat serum to HK-2 (human kidney proximal tubular cell line) cell culture and investigated whether this serum could lead renal tubular epithelial cells to mesenchymal transition and its molecular mechanism. It would help to ascertain the potential stimulation from circulation in chronic progressive kidney disease.

## MATERIALS AND METHODS

### Animals and Establishment of 5/6 Nephrectomized Rat Model

A total of 24 specified pathogen-free male Sprague Dawley (SD) rats at 3 months of age were used for this study. Each rat was housed in our animal facility under pathogen-free conditions and fed a standard laboratory diet, with free access to water. The temperature was maintained at 18-22°C with 12 h light/dark cycle. All animal procedures complied with published recommendations for use of laboratory animals by the government. On the day of the operation, the 24 SD rats were randomly divided into two groups: 5/6Nx group (5/6Nx, *n* = 12) and sham operation group (sham, *n* = 12). All rats were anesthetized with 2% pentobarbital sodium (30 mg/kg body weight, intraperitoneally). Renal mass reduction (n = 12) was obtained by ablation of two-thirds mass of the left kidney and subsequent right unilateral nephrectomy 1 week later. For the sham operation group rats a laparotomy was performed and the renal pedicle manipulated without any removal of renal mass.

After 12 weeks, serum was sampled from abdominal aorta followed by complement heat inactivation at 56°C × 30 min. Then the serum was sterilized by passing it through a 0.22 μm filter and stored at −80°C. The remnant kidney was decapsulated and divided into several parts. One part was fixed in 4% paraformaldehyde/phosphate buffered saline (PBS) and processed for histological analysis, while another part was fixed in optimum cutting temperature (OCT) compound, quickly frozen in liquid nitrogen, and stored at —80°C for immunofluorescence (IF) analysis. The remaining parts were dissected to isolate the kidney, which was quickly frozen in liquid nitrogen and stored at —80°C for protein extraction.

Serum creatinine and blood urea nitrogen (BUN) were measured with auto-biochemical analyzer (Hitachi QA36, Tokyo, Japan).

### Renal Morphologic Studies

Kidney slices 3-4 mm in thickness were fixed in buffered 10% formalin phosphate and embedded in paraffin. Sections of 2 μm thickness were prepared and stained with periodic acid-Schiff (PAS) and Masson trichrome staining. Glomerular sclerotic injury was defined as segmental accumulation of glomerular matrix and segmental or global collapse of glomerular capillaries with deposition of hyalin and adhesion of the tuft to Bowman's membrane.^16^ Tubulointerstitial injury was defined as tubular atrophy, dilation, intratubular casts, thickening of tubular basement membrane, cellular infiltration, and widening of the interstitium.

### Tissue Immunofluorescence Staining

Frozen sections of 4 μm thickness were prepared and washed with PBS twice for 10 min and preincubated in 10% casein (Vector, Burlingame, CA, USA) in PBS for 30 min. The sections were incubated in E-cadherin antibody overnight in a moisture chamber and then washed sufficiently with phosphate buffer saline-Tween (PBST) to remove unbound antibody. Next, the sections were incubated with cy3-conjugated anti-rabbit IgG antibody (Jackson ImmunoResearch, Baltimore, PA, USA) for 60 min at room temperature and washed as described for the primary antibody. The sections were mounted on glass slides and analyzed under an Olympus EX71 fluorescence microscope equipped with an Olympus DP72 digital camera (Olympus, Tokyo, Japan).

### Cell Culture and Treatment

The human kidney proximal tubular cell line (HK-2, ATCC, Manassas, VA, USA) was cultured in Dulbecco's modified Eagle medium (DMEM)/F12 (Invitrogen, Carlsbad, CA, USA), containing 2.50 g/L 4-(2-Hydroxyethyl)-1-piperazineethanesulfonic acid (HEPES) (Sigma, St. Louis, MO, USA), 1.80 g/L sodium bicarbonate (Sigma), 100 U/mL penicillin, 100 U/mL streptomycin (Invitrogen), and 10% fetal bovine serum (FBS) (Invitrogen) at 37°C in 5% CO_2_.

After digesting with 0.25% trypsin (Invitrogen), 2 × 10^5^ cells were grown in 25 cm^2^ plastic culture bottles. For experiments, HK-2 cells were cultured in DMEM/F12 10% FBS overnight and in DMEM/F12 0.1% FBS for 16 h. Then the media were changed in the following manner: DMEM/F12 10% FBS; DMEM/F12 10% sham operation serum; DMEM/F12 10% 5/6Nx rats’ 12-week serum. Cultures were continued for a further 48 h.

### Morphological Assessment of HK-2 Cells

The cells were washed twice with PBS and photographed using an Olympus EX71 fluorescence microscope equipped with an Olympus DP72 digital camera. Length/breadth ratios were used to quantify morphological changes.^17^ For each group of cultured cells, 80 cells were measured.

### Cell Immunofluorescence Microscopy

For indirect IF, cells were fixed in 4% paraformaldehyde in PBS for 10 min at 37°C, washed with PBS for 10 min, then permeabilized with ice-cold Triton buffer (0.5% Triton X-100 in 20 mM HEPES, 50 mM NaCl, 3 mM MgCl2, 300 mM sucrose) for 5 min on ice, blocked with 1% bovine serum albumin in PBS for 10 min on ice, and incubated with primary antibodies overnight in a moisture chamber. It was sufficiently washed with PBST to remove unbound antibodies. Secondary antibodies labeled with fluoresceinisothiocyanate (FITC) or cy3 (Jackson ImmunoResearch) were incubated for 60 min at room temperature and washed as with the primary antibodies. Glass cover slips carrying the treated cells were mounted with the cytosol mounting medium onto glass slides and analyzed under an Olympus EX71 fluorescence microscope equipped with an Olympus DP72 digital camera.

### Western Blotting

Kidney tissues or cultured cells were lysed in radio-immunoprecipitation assay (RIPA) buffer that was composed of 1 μg/mL leupeptin, 1 μg/mL aprotinin, and 100 μM phenylmethanesulfonyl fluoride (PMSF). All samples were spun (12,000 rpm) for 30 min at 4°C, and the protein concentration in each lysate was determined by BCA Protein Assay Kit. A total of 50 μg protein was separated by 6-10% SDS-PAGE and then transferred to a membrane which was then blocked with 5% skim milk, probed with a primary antibody at 4°C overnight, and afterward incubated with a horseradish peroxidase-conjugated secondary antibody. Antibody for E-cadherin was procured from Abcam (Cambridge, UK). Antibodies for vimentin, wnt3, β-catenin, and ZEB-1 were procured from Santa Cruz Biotechnology, Inc. (Santa Cruz, CA, USA). Antibody for β-actin was from Sigma. Finally, immunoreactive bands were visualized using enhanced chemiluminescence (ECL) reagent and exposed to X-ray film. Protein band intensities were quantified using Quantity One software (Bio-Rad, Hercules, CA, USA).

### Reverse Transcription-PCR Analysis

Total RNA was isolated using TRIzol (Invitrogen), and cDNA was synthesized with 5 mg total RNA in a reaction volume of 25 μL containing 0.5 μL oligonucleotide (dT); 20 mM dNTPs; 0.5 μL RNasin; 0.1 M DTT; 5 μL buffer; and 1 μL M-MLV reverse transcriptase (Gibco BRL, Gaithersburg, MD, USA). The final RT product mix of 1 μL was then amplified with PCR. Primers for RT-PCRwere as follows:

**Table:** 

Human E-cadherin	Sense: 5′-CGC CCT ATG ATT CTC TGC TCG-3′
	Antisense: 5′-TCG TCC TCG CCG CCT CCG TA-3′
Human vimentin	Sense: 5′-TTG AAC GCA AAG TGG AAT C-3′
	Antisense: 5′-AGG TCA GGC TTG GAA ACA-3′
Human fibronectin	Sense: 5′-TGAGAAGCCTGGGTCTCCTCC-3′
	TTGGGGAAGCTCGTCTGTCTTT-3′
Human GAPDH	Sense: 5′-AAC GAC CCC TTC ATT GAC-3′
	Antisense: 5′-TCC ACG ACA TAC TCA GCA C-3′

The annealing temperatures were 58°C for E-cadherin, fibronectin, and GAPDH and 56°C for vimentin. The number of PCR cycles used was 35 for all genes. The PCR products were resolved by electrophoresis in 1.5% agarose gel (Gibco BRL), stained with ethidium bromide, and visualized under ultraviolet light.

### Statistics

All data analyses were performed with SPSS 11.0 (SPSS Inc., Chicago, IL, USA) software. Descriptive statistics are presented as means and standard deviation (mean ± SD). Comparisons among groups were conducted with ANOVA. *p*-Value less than 0.05 was considered significant.

## RESULTS

### Serum Creatinine and Blood Urea Nitrogen Level

At the end of 12 weeks after the surgery, serum creatinine and BUN were higher in 5/6Nx rats than in sham operation rats (*p* < 0.01 vs. sham group, Table 1).

**Table 1:** Serum creatinine and blood urea nitrogen level.

Groups	Scr (μmol/L)	BUN (mmol/L)
Sham	17.60±4.51	7.76±0.87
5/6Nx	65.02±25.76*	15.24±6.45*

Notes: Sham, sham operation group; 5/6Nx, 5/6 nephrectomized group; BUN, blood urea nitrogen; Scr, Serum creatinine.

* Denotes *p* < 0.01 versus sham group.

### Renal Histopathology

**Figure 1:**
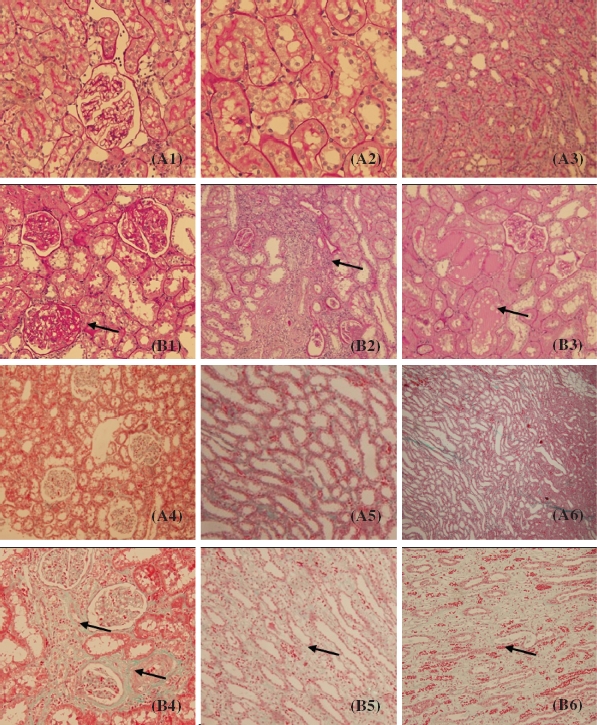
Pathological findings in 5/6 nephrectomized rats (periodic acid-Schiff (PAS) staining and Masson staining). (A1-A3) Sham group (PAS staining). (B1-B3) 5/6 nephrectomized group (PAS staining). (A4-A6) Sham group (Masson staining). (B4-B6) 5/6 nephrectomized group (Masson staining). The arrow points indicate glomerular sclerosis, inflammatory cell infiltration of the interstitium, protein casts, tubular atrophy, and interstitial fibrosis.

The histopathological alterations in the glomeruli and tubulointerstitium were evaluated by microscopical examination of the sections stained with PAS and Masson trichrome staining. Rats of sham operation groups showed normal glomerular and tubulointerstitial morphology. 5/6Nx rats showed segmental sclerosis of remnant glomeruli, enlargement of tubular lumen, protein cast, tubular atrophy, interstitial expansion accompanied by numerous infiltrations of mononuclear cells and interstitial fibrosis (Figure 1).

According to the serum creatinine, BUN level, and renal histopathology, we could define that the 5/6Nx rat models were established successfully, thus the 5/6Nx rats’ 12-week serum was qualified for the experiment.

### Expression of E-Cadherin in Renal Tubular Epithelial Cells of the Remaining Kidney

**Figure 2:**
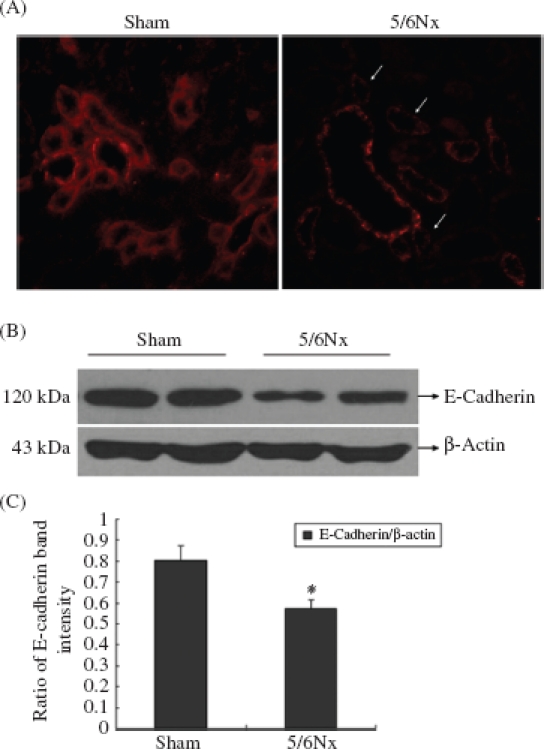
Expression of E-cadherin in kidney frozen sections and tissues. (A) The localization of E-cadherin in frozen sections was determined by IF (200x) with E-cadherin antibody (red). (B) Western blot analysis of E-cadherin in kidney tissues. Lanes 1 and 2 are sham operation group and lanes 3 and 4 are 5/6Nx group. (C) E-cadherin protein levels. Data were expressed versus β-actin and compared with ANOVA. Note: * Denotesp < 0.05 versus sham operation group.

E-Cadherin was expressed in most of the tubular epithelial cells of the rats in the sham operation group, while it was decreased significantly in the remaining kidney at 12 weeks (*p* < 0.05, Figure 2), by Western blot analyses and IF staining.

### Morphological Assessment of HK-2 Cells

**Figure 3:**
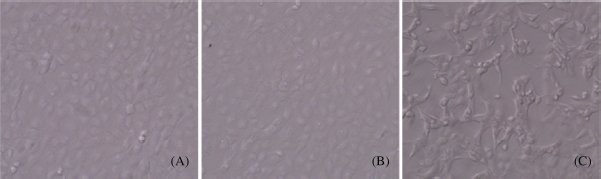
Morphological assessment of HK-2 cells. (A) 10% FBS group (100x); (B) 10% sham serum group (100x); (C) 10% 5/6 nephrectomized rat serum group (100x).

To investigate whether the 5/6Nx rat serum could influence the morphology of HK-2 cells, microscope was used; 10% FBS and 10% sham serum groups showed typical cobblestone morphology of epithelial cells, while the cell morphology changes after they were treated for 48 h with 10% 5/6 nephrectomized rat serum (5/6Nx). Cells became more elongated in shape, disassociated from neighboring cells, and lost their cobblestone monolayer pattern (Figure 3). Length/ breadth ratios for 5/6Nx rat serum group (3.97 ± 1.04) were significantly greater than for FBS (1.35 ± 0.36) or sham operation serum groups (1.39 ± 0.41) (*p* < 0.01, Table 2).

**Table 2:** Effect of 5/6 nephrectomized rat serum on morphology of HK-2 cells (length/width ratio).

Group	Cell length/width
FBS	1.35 ± 0.36
Sham serum	1.39 ± 0.41
5/6Nx serum	3.97 ± 1.04*

Notes: Sham, sham operation group; 5/6Nx, 5/6 nephrectomized group.

* Denotes *p* < 0.01 versus 10% sham operation serum group.

### Effect of 5/6 Nephrectomized Rat Serum on Tubular Epithelial-to-Mesenchymal Transition

**Figure 4:**
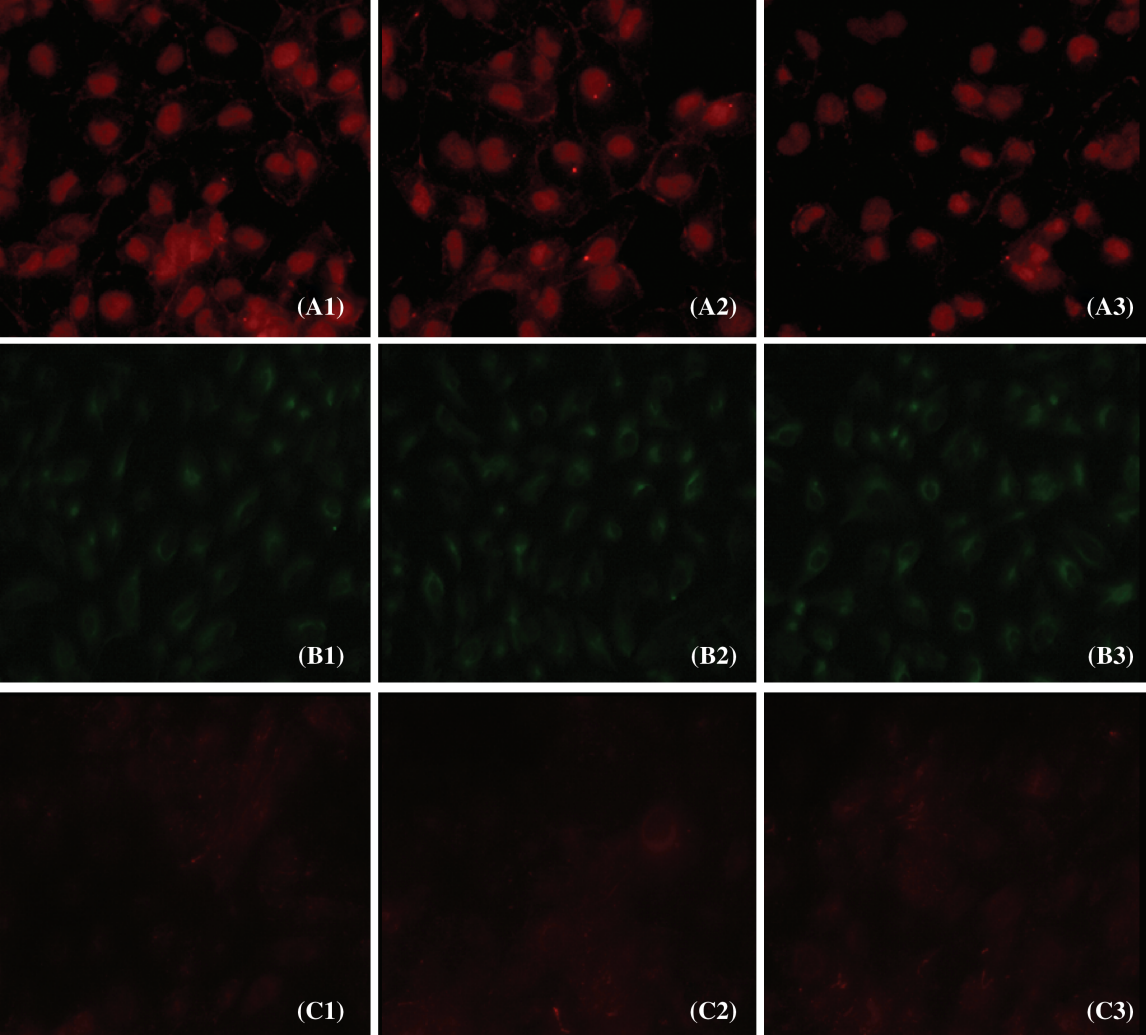
Expression of E-cadherin, vimentin, and fibronectin protein in cultured cells. The localization of E-cadherin, vimentin, and fibronectin was determined by IF (400x) with E-cadherin antibody (red) (A1-A3), vimentin antibody (green) (B1-B3), and fibronectin antibody (red) (C1-C3).

**Figure 5:**
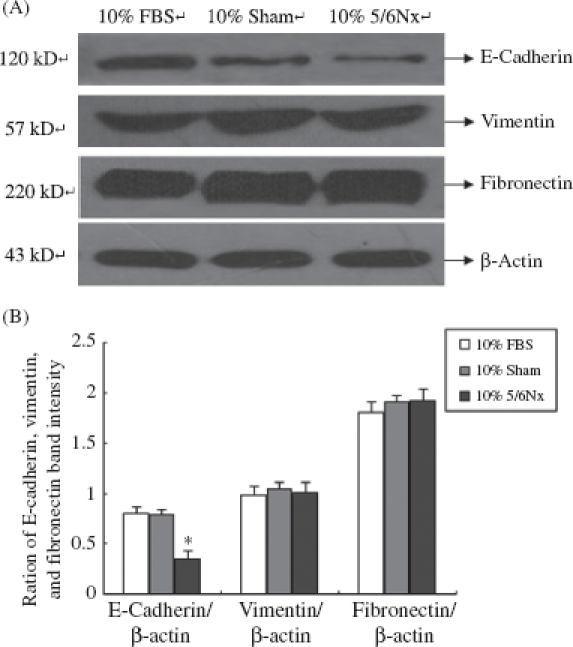
Expression of E-cadherin, vimentin, and fibronectin in HK-2 cells. (A) Western blot of E-cadherin, vimentin, and fibronectin. Lanes 1-3 are 10% FBS group, 10% sham operation serum group, and 10% 5/6 nephrectomized rat serum group, respectively. (B) E-cadherin, vimentin, and fibronectin protein levels. Data were expressed versus β-actin and compared with ANOVA. Notes: The experiment was repeated three times with similar results. * Denotes *p* < 0.05 versus 10% sham operation serum group.

**Figure 6:**
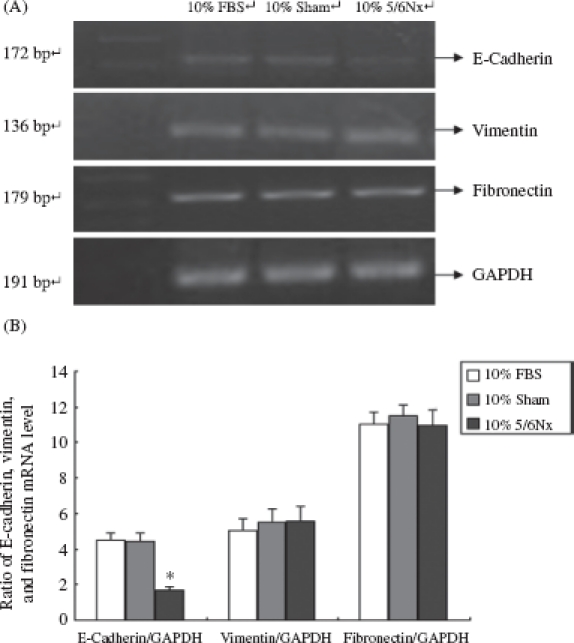
E-Cadherin, vimentin, and fibronectin mRNA in HK-2 cells. (A) Total RNA isolated from rats at different ages was analyzed with RT-PCR. Lanes 1-4 are marker (DL2000), 10% FBS group, 10% sham operation serum group, and 10% 5/6 nephrectomized rat serum group, respectively. (B) Expression analysis of E-cadherin, vimentin, and fibronectin mRNA abundance with ANOVA. Notes: The experiment was repeated three times with similar results. * Denotes *p* < 0.05 versus 10% sham operation serum group.

IF, Western blot, and RT-PCR analysis were performed in 10% FBS, 10% sham serum, and 10% 5/6Nx serum group cells to detect the expression of E-cadherin, vimentin, and fibronectin in HK-2 cells (Figures 4-6).

**Figure 7:**
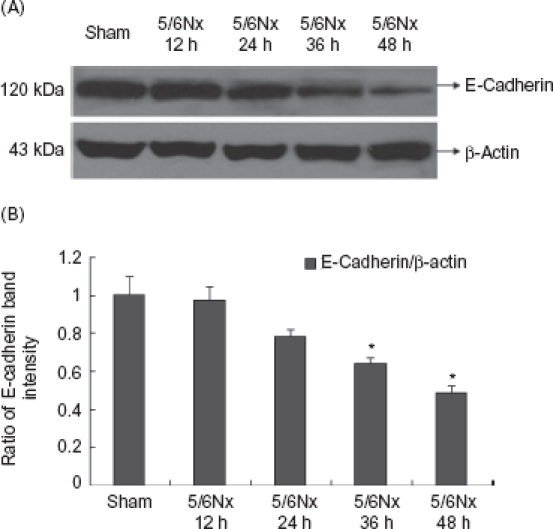
Expression of E-cadherin at different time points of HK-2 cells. (A) Western blot analysis of E-cadherin in cultured cells at different time points. (B) E-Cadherin protein levels. Data were expressed versus β-actin and compared with ANOVA. * Denotes *p* < 0.05 versus sham operation serum group.

As shown in Figures 4-6, the 5/6Nx rat serum could suppress HK-2 cells expressing protein and mRNA of E-cadherin (*p* < 0.05), though it seemed there was no significant effect on the expression of vimentin and fibronectin. We found that the 5/6Nx rat serum had a time-dependent effect on the expression of E-cadherin (Figure 7).

### Effect of 5/6 Nephrectomized Rat Serum on the Expression of ZEB1, β-Catenin, and wnt3 in HK-2 Cells

**Figure 8:**
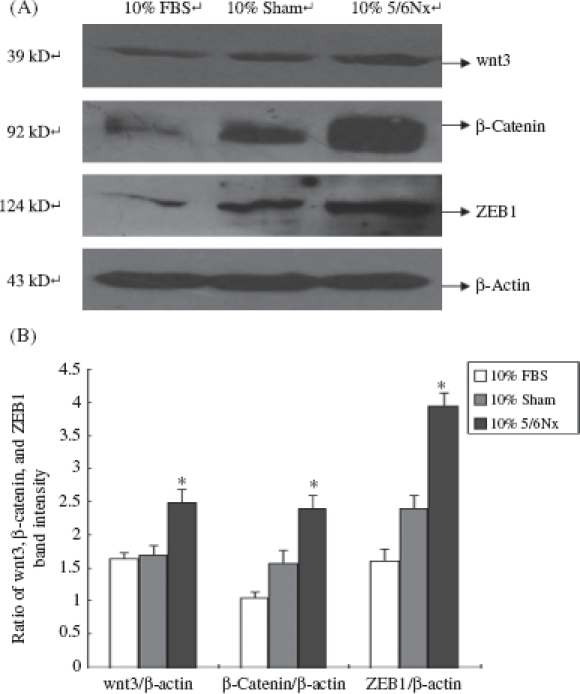
Expression of wnt3, β-catenin, and ZEB1 in HK-2 cells. (A) Western blot analysis of wnt3, β-catenin, and ZEB1. Lanes 1—3 are 10% FBS group, 10% sham operation serum group, and 10% 5/6 nephrectomized rat serum group, respectively. (B) wnt3, β-catenin, and ZEB1 protein levels. Data were expressed versus β-actin and compared with ANOVA. Notes: The experiment was repeated three times with similar results. * Denotes *p* < 0.05 versus 10% sham operation serum group.

The expressions of ZEB1, β-catenin, and wnt3 were detected by Western blot analysis. Our results showed that compared with 10% FBS and 10% sham serum groups, HK-2 cells incubated in 10% 5/6 nephrectomized rat serum lead to the activation of ZEB1, β-catenin, and wnt3 (Figure 8).

## DISCUSSION

In this study, the effect of 5/6Nx rat serum on tubular EMT in vitro was detected. Our study demonstrated that E-cadherin expression was decreased significantly in the remaining kidney at 12 weeks. With the 5/6Nx rats’ 12-week serum treated for 48 h, E-cadherin mRNA and protein levels of HK-2 cells were significantly down-regulated, though it seemed there was no significant effect on the expression of vimentin and fibronectin. We provided the evidence that 5/6Nx rat serum contributed to the activation of ZEB1 and led to the activation of Wnt signaling pathway.

In CKD patients, with the decrease in glomerular filtration rate, the excretion of metabolites failed. The body also underwent oxidative stress, carboxyl shock, micro-inflammation, and other pathological and physiological changes. As a result, some metabolic toxins were accumulated in the body, including advanced glycation products,^18^ advanced oxidation protein products,^11^ homocysteine,^12^ and so on. The endocrine system in CKD patients also turns up some abnormalities. Circulating RAS was active. Ang II hemodynamically could significantly increase blood pressure, glomerular capillary pressure, and the permeability of filtration membrane, which promoted glomerular sclerosis and renal interstitial fibrosis.^13^ It also had non-hemodynamic effects, like increasing the expression of TGF-β, chemokines, and the activity of macrophage. It also promoted the deposition of extracellular matrix, cell proliferation, and tissue remodeling.^19^

Accumulation of metabolic toxins from peritubular capillaries could stimulate renal tubular epithelial cells and the abnormal endocrine system could affect the adherence function of renal tubular epithelial cells through glomerular and peritubular capillaries. In our study, we first demonstrated that E-cadherin expression was decreased significantly in the remaining kidney at 12 weeks. Then we confirmed that 5/6Nx rats’ 12-week serum, which was similar to the pathogenic serum of CKD patients, could suppress the expression of intercellular adhesion junction protein E-cadherin in HK-2 cells. In the present technology, we can hardly clarify the constituents of the 5/6Nx rats’ 12-week serum. However, the mechanism we demonstrated should provide us a new vision for the prevention and treatment of CKD.

Downregulation of E-cadherin marks the initiation of EMT.^20^ The most important event of EMT is the loss of E-cadherin. E-Cadherin is a hallmark of epithelial cell layers and is localized at the basolateral membrane in adherens junctions. The core molecule of adherens junctions, E-cadherin connects neighboring epithelial cells by calcium-dependent homotypic interactions of its extracellular tail.^21^

We found that 5/6Nx rat serum could downregulate E-cadherin mRNA and protein expressions in HK-2 cells, considering this pathogenic serum regulated the expression of E-cadherin in transcriptional control. ZEB1 is a member of the ZEB family, which is widely known as the member of E-cadherin transcriptional repressors.^22^-^24^

ZEB1 can interact with DNA through the simultaneous binding of the two zinc-finger domains to high-affinity binding sites composed of bipartite E-boxes (CACCT and CACCTG), as is found in the E-cadherin promoter, and then suppress the synthesis of the cell-cell adhesion protein.^25^ ZEB1 also promotes EMT by repressing expression of basement membrane components and cell polarity proteins.^26^ Our Western blot analysis results indicated that ZEB1 was significantly increased in 5/6Nx rat serum group. We provided the evidence that the activation of ZEB1 contributed to the downregulation of E-cadherin.

We found that the 5/6Nx rat serums have no significant effect on the expression of vimentin and fibronectin in HK-2 cells. However, downregulation of E-cadherin has several important consequences which are of direct relevance to EMT. The cytoplasmic part of E-cadherin interacts with the other components of adherens junctions, in particular β-catenin.^27^ The consequence of a loss of E-cadherin is an impairment of cell-cell adhesion, which allows detachment of cells. E-Cadherin-mediated sequestering of β-catenin in the cytoplasm is abolished and leads to nuclear localization of β-catenin. β-Catenin has also been demonstrated to play an important role in the induction of EMT. As a result, β-catenin localizes to the nucleus and feeds into the Wnt signaling pathway by activating transcriptional regulation through LEF/TCF4 (lymphoid enhancer-binding factor/T-cell factor-4).^28^

Our study showed that the expressions of β-catenin and wnt3 were all upregulated. It indicated that although the 5/6Nx rat serum could not lead the tubular epithelial cells to mesenchymal transition, it could suppress the expression of E-cadherin. The loss of E-cadherin led β-catenin to localize to the cytoplasm and nucleus, while nuclear localization of β-catenin fed into the Wnt signaling pathway. Wnt signaling is also linked to EMT, by direct activation of snail or by indirect activation of ZEB 1 via other WNT target genes, for example, COX2^29^ or IGF1.^30^ Taken together, we found that the 5/6Nx rat serum could lead the tubular cell into this pathological loop involving E-cadherin, ZEB1, and β-catenin. This pathological loop is the crucial step of EMT.^31^ Interference with these crosstalks might offer novel therapeutic opportunities in CKD in the future.

## References

[1] Nangaku M (2004). Mechanisms of tubulointerstitial injury in the kidney: Final common pathways to end-stage renal failure. Intern Med.

[2] Zeisberg M, Kaluri R (2004). The role of epithelial-to-mesenchymal transition in renal fibrosis. J Mol Med.

[3] Venkov CD, Link AJ, Jennings JL (2007). A proximal activator of transcription in epithelial-mesenchymal transition. J Clin Invest.

[4] Yang J, Liu Y (2001). Dissection of key events in tubular epithelial to myofibroblast transition and its implications in renal interstitial fibrosis. Am J Pathol.

[5] Lan HY (2003). Tubular epithelial-myofibroblast transdifferentiation mechanisms in proximal tubule cells. Curr Opin Nephrol Hypertens.

[6] Fan JM, Huang XR, Ng YY (2001). Interleukin-1 induces tubular epithelial-myofibroblast transdifferentiation through a transforming growth factor-beta 1-dependent mechanism in vitro. Am J Kidney Dis.

[7] Strutz F, Zeisberg M, Ziyadeh FN (2002). Role of basic fibrob-last growth factor-2 in epithelial-mesenchymal transformation. Kidney Int.

[8] Okada H, Danoff TM, Kalluri R, Neilson EG (1997). Early role of FSP1 in epithelial-mesenchymal transformation. Am J Physiol.

[9] Ha H, Lee HB (2003). Reactive oxygen species and matrix remodeling in diabetic kidney. J Am Soc Nephrol.

[10] Guarino M, Tosoni A, Nebuloni M (2009). Direct contribution of epithelium to organ fibrosis: Epithelial-mesenchymal transition. HumPathol.

[11] Li HY, Hou FF, Zhang X (2007). Advanced oxidation protein products accelerate renal fibrosis in a remnant kidney model. JAm Soc Nephrol.

[12] Kumagai H, Katoh S, Hirosawa K, Kimura M, Hishida A, Ikegaya N (2002). Renal tubulointerstitial injury in weaning rats with hyperhomocysteinemia. Kidney Int.

[13] Remuzzi G, Perico N, Macia M, Ruggenenti P (2005). The role of renin-angiotensin-aldosterone system in the progression of chronic kidney disease. Kidney Int.

[14] Kliem V, Johnson RJ, Alpers CE (1996). Mechanisms involved in the pathogenesis of tubulointerstitial fibrosis in 5/6-nephrectomized rats. Kidney Int.

[15] Yan-chun S, Xiao Y, Qiong-xia C (2005). Renal pathological changes in rat with 5/6 nephrectomy. Shanghai Lab Animal Sci.

[16] Floege J, Hackmann B, Kliem V (1997). Age-related glomeru-losclerosis and interstitial fibrosis in Milan normotensive rats: Apodocyte disease. Kidney Int.

[17] Copeland JW, Beaumont BW, Merrilees MJ, Pilmore HL (2007). Epithelial-to-mesenchymal transition of human proximal tubular epithelial cells: Effects of rapamycin, mycopheno-late, cyclosporin, azathioprine, and methylprednisolone. Transplantation.

[18] Feng JX, Hou FF, Liang M (2007). Restricted intake of dietary advanced glycation end products retards renal progression in the remnant kidney model. Kidney Int.

[19] Aros C, Remuzzi G (2002). The renin-angiotensin system in progression, remission and regression of chronic nephropathies. JHypertens.

[20] Yook JI, Li XY, Ota I, Fearon ER, Weiss SJ (2005). Wnt-dependent regulation of the E-cadherin repressor snail. J Biol Chem.

[21] Perez-Moreno M, Jamora C, Fuchs E (2003). Sticky business: Orchestrating cellular signals at adherens junctions. Cell.

[22] Shirakihara T, Saitoh M, Miyazono K (2007). Differential regulation of epithelial and mesenchymal markers by deltaEF1 proteins in epithelial mesenchymal transition induced by TGF-beta. MolBiol Cell.

[23] Spaderna S, Schmalhofer O, Wahlbuhl M (2008). The transcriptional repressor ZEB1 promotes metastasis and loss of cell polarity in cancer. Cancer Res.

[24] Aigner K, Dampier B, Descovich L (2007). The transcription factor ZEB1 (deltaEF1) promotes tumour cell dedifferentiation by repressing master regulators of epithelial polarity. Oncogene.

[25] Remacle JE, Kraft H, Lerchner W (1999). New mode of DNA binding of multi-zinc finger transcription factors: DeltaEF1 family members bind with two hands to two target sites. EMBOJ.

[26] Comijn J, Berx G, Vermassen P (2001). The two-handed E box binding zinc finger protein SIP1 downregulates E-cadherin and induces invasion. Mol Cell.

[27] Perez-Moreno M, Fuchs E (2006). Catenins: Keeping cells from getting their signals crossed. Dev Cell.

[28] Thiery JP, Sleeman JP (2006). Complex networks orchestrate epithelial-mesenchymal transitions. Nat Rev Mol Cell Biol.

[29] Longo KA, Kennell JA, Ochocinska MJ, Ross SE, Wright WS, MacDougald OA (2002). Wnt signaling protects 3T3-L1 preadipocytes from apoptosis through induction of insulin-like growth factors. J Biol Chem.

[30] Shao J, Jung C, Liu C, Sheng H (2005). Prostaglandin E2 stimulates the beta-catenin/T cell factor-dependent transcription in colon cancer. J Biol Chem.

[31] Schmalhofer O, Brabletz S, Brabletz T (2009). E-Cadherin, beta-catenin, and ZEB1 in malignant progression of cancer. Cancer Metastasis Rev.

